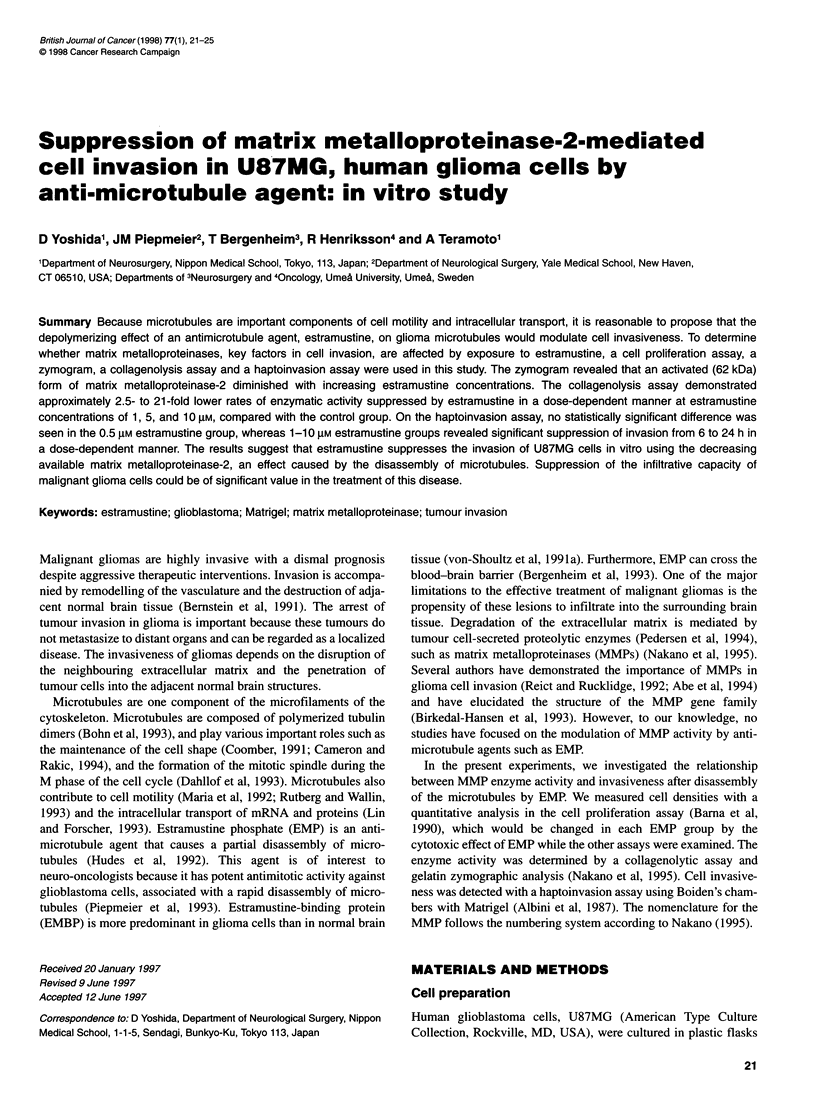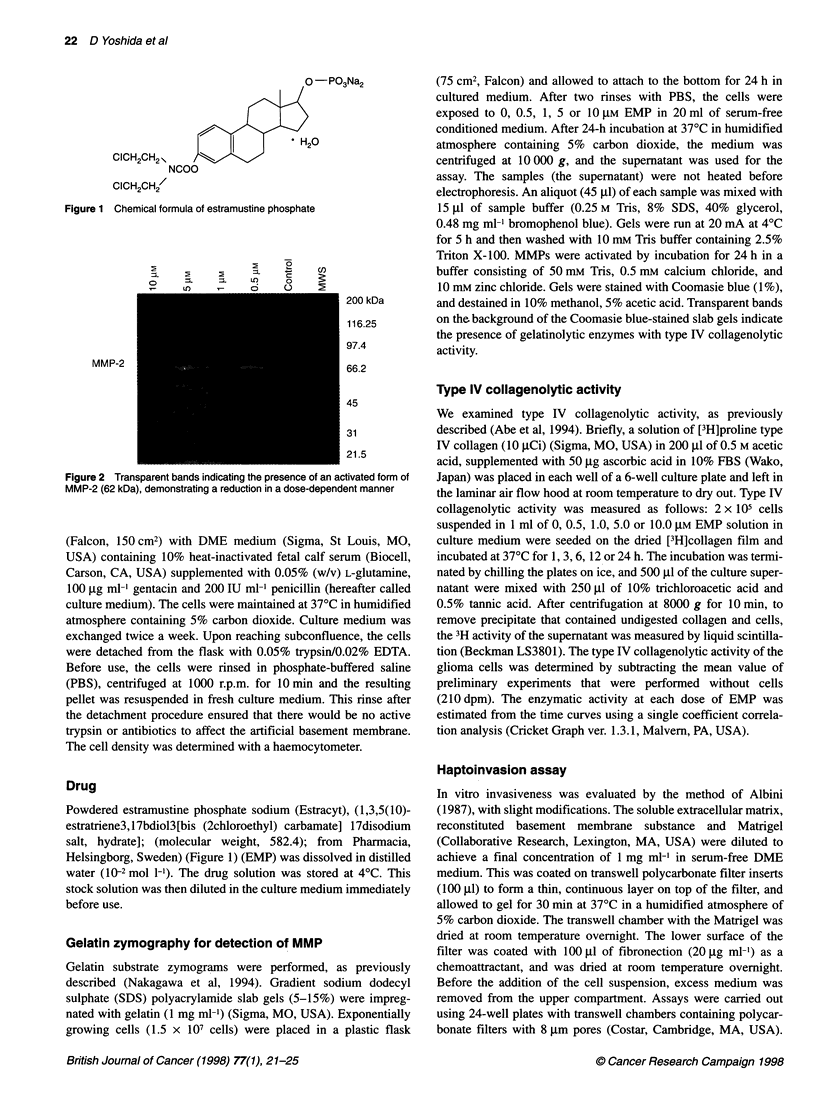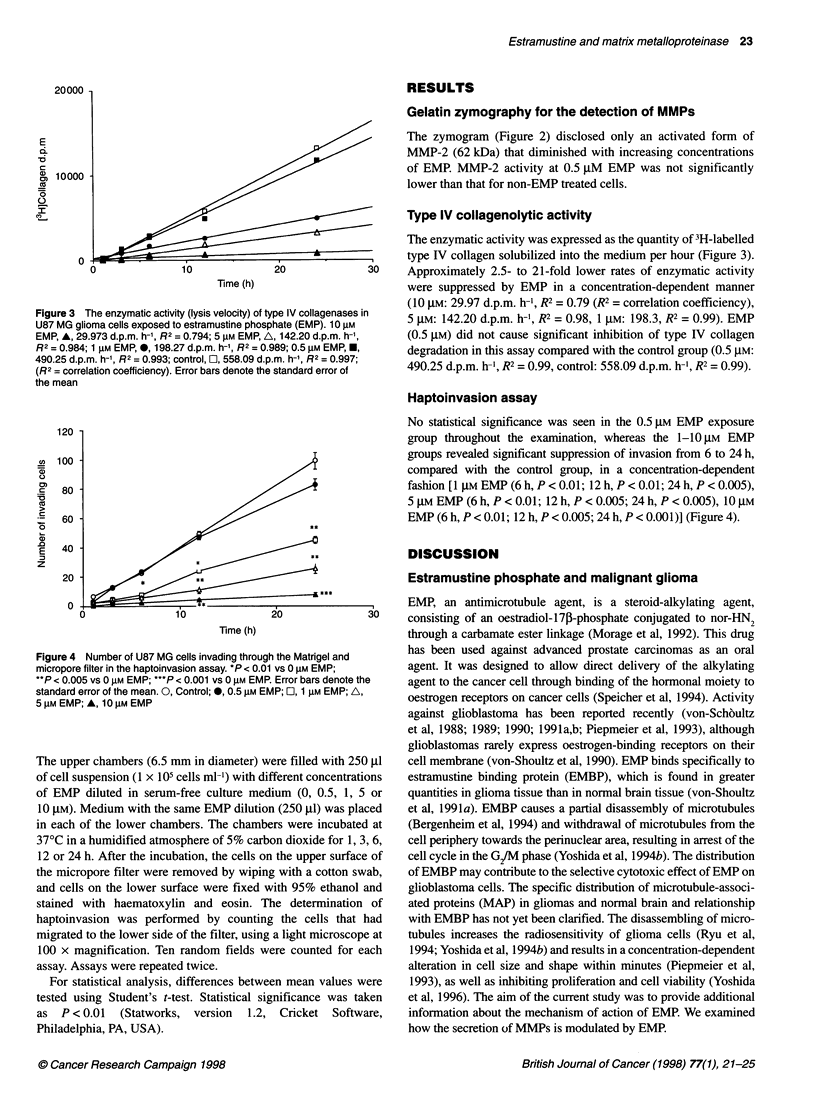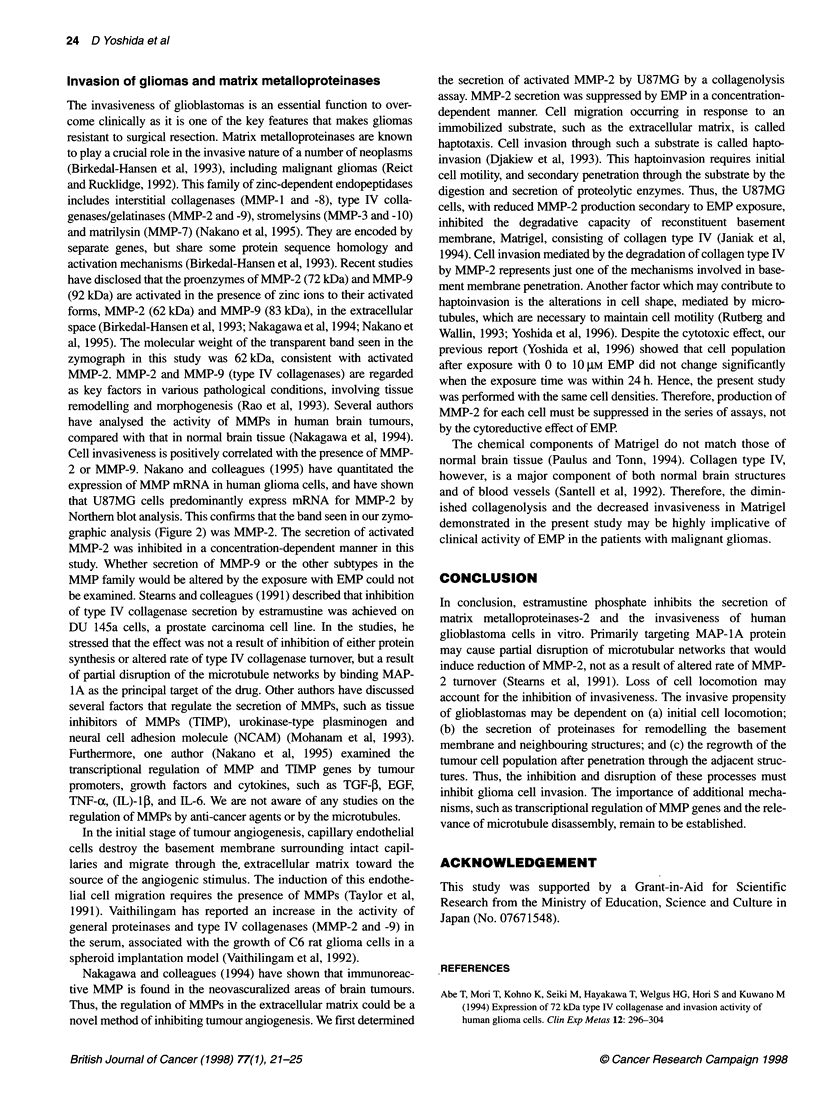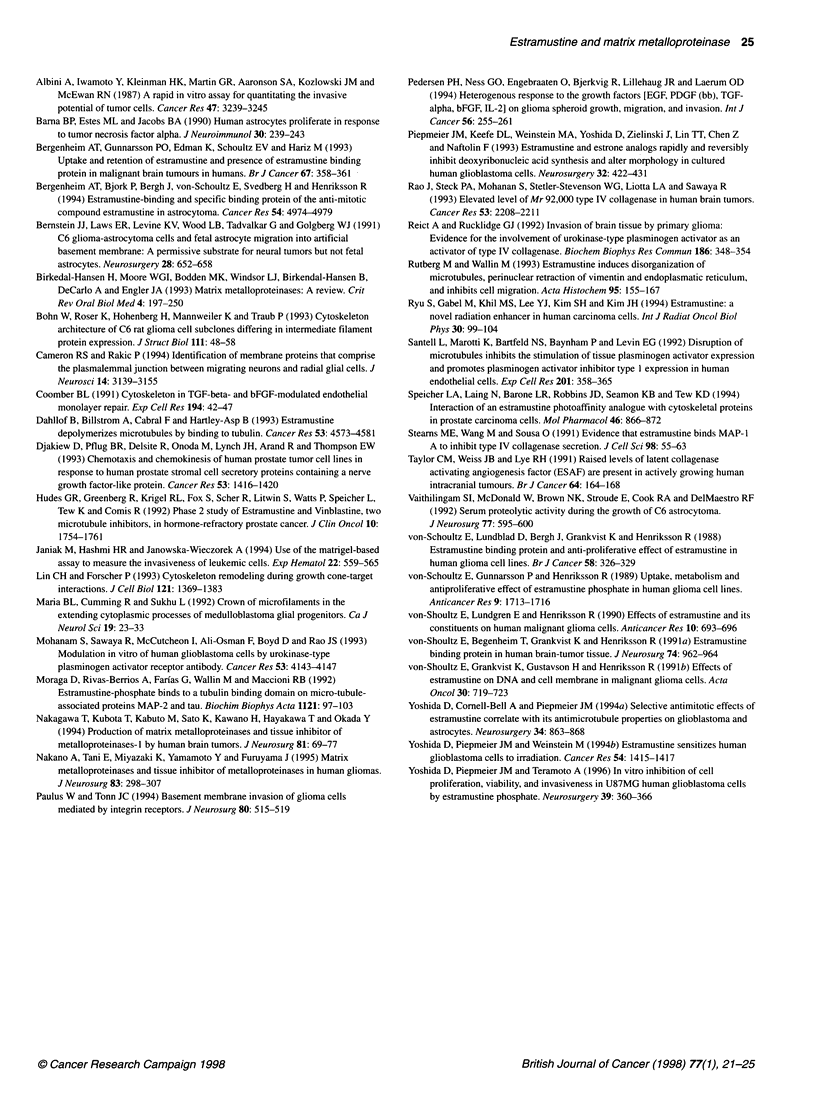# Suppression of matrix metalloproteinase-2-mediated cell invasion in U87MG, human glioma cells by anti-microtubule agent: in vitro study.

**DOI:** 10.1038/bjc.1998.4

**Published:** 1998

**Authors:** D. Yoshida, J. M. Piepmeier, T. Bergenheim, R. Henriksson, A. Teramoto

**Affiliations:** Department of Neurosurgery, Nippon Medical School, Tokyo, Japan.

## Abstract

**Images:**


British Journal of Cancer (1998) 77(1), 21–25
© 1998 Cancer Research Campaign

### Suppression of matrix metalloproteinase-2-mediated cell invasion in U87MG, human glioma cells by anti-microtubule agent: in vitro study

**D Yoshida[1], JM Piepmeier[2], T Bergenheim[3], R Henriksson[4] and A Teramoto[1]**

[1]Department of Neurosurgery, Nippon Medical School, Tokyo, 113, Japan; [2]Department of Neurological Surgery, Yale Medical School, New Haven, CT 06510, USA; Departments of [3]Neurosurgery and [4]Oncology, Umeå University, Umeå, Sweden

**Summary** Because microtubules are important components of cell motility and intracellular transport, it is reasonable to propose that the depolymerizing effect of an antimicrotubule agent, estramustine, on glioma microtubules would modulate cell invasiveness. To determine whether matrix metalloproteinases, key factors in cell invasion, are affected by exposure to estramustine, a cell proliferation assay, a zymogram, a collagenolysis assay and a haptoinvasion assay were used in this study. The zymogram revealed that an activated (62 kDa) form of matrix metalloproteinase-2 diminished with increasing estramustine concentrations. The collagenolysis assay demonstrated approximately 2.5- to 21-fold lower rates of enzymatic activity suppressed by estramustine in a dose-dependent manner at estramustine concentrations of 1, 5, and 10 μM, compared with the control group. On the haptoinvasion assay, no statistically significant difference was seen in the 0.5 μM estramustine group, whereas 1–10 μM estramustine groups revealed significant suppression of invasion from 6 to 24 h in a dose-dependent manner. The results suggest that estramustine suppresses the invasion of U87MG cells in vitro using the decreasing available matrix metalloproteinase-2, an effect caused by the disassembly of microtubules. Suppression of the infiltrative capacity of malignant glioma cells could be of significant value in the treatment of this disease.

**Keywords:** estramustine; glioblastoma; Matrigel; matrix metalloproteinase; tumour invasion

Malignant gliomas are highly invasive with a dismal prognosis despite aggressive therapeutic interventions. Invasion is accompanied by remodelling of the vasculature and the destruction of adjacent normal brain tissue (Bernstein et al, 1991). The arrest of tumour invasion in glioma is important because these tumours do not metastasize to distant organs and can be regarded as a localized disease. The invasiveness of gliomas depends on the disruption of the neighbouring extracellular matrix and the penetration of tumour cells into the adjacent normal brain structures.

Microtubules are one component of the microfilaments of the cytoskeleton. Microtubules are composed of polymerized tubulin dimers (Bohn et al, 1993), and play various important roles such as the maintenance of the cell shape (Coomber, 1991; Cameron and Rakic, 1994), and the formation of the mitotic spindle during the M phase of the cell cycle (Dahllof et al, 1993). Microtubules also contribute to cell motility (Maria et al, 1992; Rutberg and Wallin, 1993) and the intracellular transport of mRNA and proteins (Lin and Forscher, 1993). Estramustine phosphate (EMP) is an antimicrotubule agent that causes a partial disassembly of microtubules (Hudes et al, 1992). This agent is of interest to neuro-oncologists because it has potent antimitotic activity against glioblastoma cells, associated with a rapid disassembly of microtubules (Piepmeier et al, 1993). Estramustine-binding protein (EMBP) is more predominant in glioma cells than in normal brain tissue (von-Shoultz et al, 1991a). Furthermore, EMP can cross the blood–brain barrier (Bergenheim et al, 1993). One of the major limitations to the effective treatment of malignant gliomas is the propensity of these lesions to infiltrate into the surrounding brain tissue. Degradation of the extracellular matrix is mediated by tumour cell-secreted proteolytic enzymes (Pedersen et al, 1994), such as matrix metalloproteinases (MMPs) (Nakano et al, 1995). Several authors have demonstrated the importance of MMPs in glioma cell invasion (Reict and Rucklidge, 1992; Abe et al, 1994) and have elucidated the structure of the MMP gene family (Birkedal-Hansen et al, 1993). However, to our knowledge, no studies have focused on the modulation of MMP activity by antimicrotubule agents such as EMP.

In the present experiments, we investigated the relationship between MMP enzyme activity and invasiveness after disassembly of the microtubules by EMP. We measured cell densities with a quantitative analysis in the cell proliferation assay (Barna et al, 1990), which would be changed in each EMP group by the cytotoxic effect of EMP while the other assays were examined. The enzyme activity was determined by a collagenolytic assay and gelatin zymographic analysis (Nakano et al, 1995). Cell invasiveness was detected with a haptoinvasion assay using Boiden's chambers with Matrigel (Albini et al, 1987). The nomenclature for the MMP follows the numbering system according to Nakano (1995).

*Received 20 January 1997*
*Revised 9 June 1997*
*Accepted 12 June 1997*

*Correspondence to:* D Yoshida, Department of Neurological Surgery, Nippon Medical School, 1-1-5, Sendagi, Bunkyo-Ku, Tokyo 113, Japan

### MATERIALS AND METHODS

### Cell preparation

Human glioblastoma cells, U87MG (American Type Culture Collection, Rockville, MD, USA), were cultured in plastic flasks

**Figure 1** Chemical formula of estramustine phosphate

**Figure 2** Transparent bands indicating the presence of an activated form of MMP-2 (62 kDa), demonstrating a reduction in a dose-dependent manner

(Falcon, 150 cm$^2$) with DME medium (Sigma, St Louis, MO, USA) containing 10% heat-inactivated fetal calf serum (Biocell, Carson, CA, USA) supplemented with 0.05% (w/v) L-glutamine, 100 μg ml$^{-1}$ gentacin and 200 IU ml$^{-1}$ penicillin (hereafter called culture medium). The cells were maintained at 37°C in humidified atmosphere containing 5% carbon dioxide. Culture medium was exchanged twice a week. Upon reaching subconfluence, the cells were detached from the flask with 0.05% trypsin/0.02% EDTA. Before use, the cells were rinsed in phosphate-buffered saline (PBS), centrifuged at 1000 r.p.m. for 10 min and the resulting pellet was resuspended in fresh culture medium. This rinse after the detachment procedure ensured that there would be no active trypsin or antibiotics to affect the artificial basement membrane. The cell density was determined with a haemocytometer.

### Drug

Powdered estramustine phosphate sodium (Estracyt), (1,3,5(10)-estratriene3,17bdiol3[bis (2chloroethyl) carbamate] 17disodium salt, hydrate]; (molecular weight, 582.4); from Pharmacia, Helsingborg, Sweden) (Figure 1) (EMP) was dissolved in distilled water (10$^{-2}$ mol l$^{-1}$). The drug solution was stored at 4°C. This stock solution was then diluted in the culture medium immediately before use.

### Gelatin zymography for detection of MMP

Gelatin substrate zymograms were performed, as previously described (Nakagawa et al, 1994). Gradient sodium dodecyl sulphate (SDS) polyacrylamide slab gels (5–15%) were impregnated with gelatin (1 mg ml$^{-1}$) (Sigma, MO, USA). Exponentially growing cells (1.5 × 10$^7$ cells) were placed in a plastic flask (75 cm$^2$, Falcon) and allowed to attach to the bottom for 24 h in cultured medium. After two rinses with PBS, the cells were exposed to 0, 0.5, 1, 5 or 10 μM EMP in 20 ml of serum-free conditioned medium. After 24-h incubation at 37°C in humidified atmosphere containing 5% carbon dioxide, the medium was centrifuged at 10 000 *g*, and the supernatant was used for the assay. The samples (the supernatant) were not heated before electrophoresis. An aliquot (45 μl) of each sample was mixed with 15 μl of sample buffer (0.25 M Tris, 8% SDS, 40% glycerol, 0.48 mg ml$^{-1}$ bromophenol blue). Gels were run at 20 mA at 4°C for 5 h and then washed with 10 mM Tris buffer containing 2.5% Triton X-100. MMPs were activated by incubation for 24 h in a buffer consisting of 50 mM Tris, 0.5 mM calcium chloride, and 10 mM zinc chloride. Gels were stained with Coomasie blue (1%), and destained in 10% methanol, 5% acetic acid. Transparent bands on the background of the Coomasie blue-stained slab gels indicate the presence of gelatinolytic enzymes with type IV collagenolytic activity.

### Type IV collagenolytic activity

We examined type IV collagenolytic activity, as previously described (Abe et al, 1994). Briefly, a solution of [$^3$H]proline type IV collagen (10 μCi) (Sigma, MO, USA) in 200 μl of 0.5 M acetic acid, supplemented with 50 μg ascorbic acid in 10% FBS (Wako, Japan) was placed in each well of a 6-well culture plate and left in the laminar air flow hood at room temperature to dry out. Type IV collagenolytic activity was measured as follows: 2 × 10$^5$ cells suspended in 1 ml of 0, 0.5, 1.0, 5.0 or 10.0 μM EMP solution in culture medium were seeded on the dried [$^3$H]collagen film and incubated at 37°C for 1, 3, 6, 12 or 24 h. The incubation was terminated by chilling the plates on ice, and 500 μl of the culture supernatant were mixed with 250 μl of 10% trichloroacetic acid and 0.5% tannic acid. After centrifugation at 8000 *g* for 10 min, to remove precipitate that contained undigested collagen and cells, the $^3$H activity of the supernatant was measured by liquid scintillation (Beckman LS3801). The type IV collagenolytic activity of the glioma cells was determined by subtracting the mean value of preliminary experiments that were performed without cells (210 dpm). The enzymatic activity at each dose of EMP was estimated from the time curves using a single coefficient correlation analysis (Cricket Graph ver. 1.3.1, Malvern, PA, USA).

### Haptoinvasion assay

In vitro invasiveness was evaluated by the method of Albini (1987), with slight modifications. The soluble extracellular matrix, reconstituted basement membrane substance and Matrigel (Collaborative Research, Lexington, MA, USA) were diluted to achieve a final concentration of 1 mg ml$^{-1}$ in serum-free DME medium. This was coated on transwell polycarbonate filter inserts (100 μl) to form a thin, continuous layer on top of the filter, and allowed to gel for 30 min at 37°C in a humidified atmosphere of 5% carbon dioxide. The transwell chamber with the Matrigel was dried at room temperature overnight. The lower surface of the filter was coated with 100 μl of fibronection (20 μg ml$^{-1}$) as a chemoattractant, and was dried at room temperature overnight. Before the addition of the cell suspension, excess medium was removed from the upper compartment. Assays were carried out using 24-well plates with transwell chambers containing polycarbonate filters with 8 μm pores (Costar, Cambridge, MA, USA).

© Cancer Research Campaign 1998

**Figure 3** The enzymatic activity (lysis velocity) of type IV collagenases in U87 MG glioma cells exposed to estramustine phosphate (EMP). 10 μM EMP, ▲, 29.973 d.p.m. $h^{-1}$, $R^2$ = 0.794; 5 μM EMP, △, 142.20 d.p.m. $h^{-1}$, $R^2$ = 0.984; 1 μM EMP, ●, 198.27 d.p.m. $h^{-1}$, $R^2$ = 0.989; 0.5 μM EMP, ■, 490.25 d.p.m. $h^{-1}$, $R^2$ = 0.993; control, □, 558.09 d.p.m. $h^{-1}$, $R^2$ = 0.997; ($R^2$ = correlation coefficiency). Error bars denote the standard error of the mean

**Figure 4** Number of U87 MG cells invading through the Matrigel and micropore filter in the haptoinvasion assay. *$P$ < 0.01 vs 0 μM EMP; **$P$ < 0.005 vs 0 μM EMP; ***$P$ < 0.001 vs 0 μM EMP. Error bars denote the standard error of the mean. ○, Control; ●, 0.5 μM EMP; □, 1 μM EMP; △, 5 μM EMP; ▲, 10 μM EMP

The upper chambers (6.5 mm in diameter) were filled with 250 μl of cell suspension (1 × $10^5$ cells $ml^{-1}$) with different concentrations of EMP diluted in serum-free culture medium (0, 0.5, 1, 5 or 10 μM). Medium with the same EMP dilution (250 μl) was placed in each of the lower chambers. The chambers were incubated at 37°C in a humidified atmosphere of 5% carbon dioxide for 1, 3, 6, 12 or 24 h. After the incubation, the cells on the upper surface of the micropore filter were removed by wiping with a cotton swab, and cells on the lower surface were fixed with 95% ethanol and stained with haematoxylin and eosin. The determination of haptoinvasion was performed by counting the cells that had migrated to the lower side of the filter, using a light microscope at 100 × magnification. Ten random fields were counted for each assay. Assays were repeated twice.

For statistical analysis, differences between mean values were tested using Student's $t$-test. Statistical significance was taken as $P$ < 0.01 (Statworks, version 1.2, Cricket Software, Philadelphia, PA, USA).

### RESULTS

### Gelatin zymography for the detection of MMPs

The zymogram (Figure 2) disclosed only an activated form of MMP-2 (62 kDa) that diminished with increasing concentrations of EMP. MMP-2 activity at 0.5 μM EMP was not significantly lower than that for non-EMP treated cells.

### Type IV collagenolytic activity

The enzymatic activity was expressed as the quantity of $^3$H-labelled type IV collagen solubilized into the medium per hour (Figure 3). Approximately 2.5- to 21-fold lower rates of enzymatic activity were suppressed by EMP in a concentration-dependent manner (10 μM: 29.97 d.p.m. $h^{-1}$, $R^2$ = 0.79 ($R^2$ = correlation coefficiency), 5 μM: 142.20 d.p.m. $h^{-1}$, $R^2$ = 0.98, 1 μM: 198.3, $R^2$ = 0.99). EMP (0.5 μM) did not cause significant inhibition of type IV collagen degradation in this assay compared with the control group (0.5 μM: 490.25 d.p.m. $h^{-1}$, $R^2$ = 0.99, control: 558.09 d.p.m. $h^{-1}$, $R^2$ = 0.99).

### Haptoinvasion assay

No statistical significance was seen in the 0.5 μM EMP exposure group throughout the examination, whereas the 1–10 μM EMP groups revealed significant suppression of invasion from 6 to 24 h, compared with the control group, in a concentration-dependent fashion [1 μM EMP (6 h, $P$ < 0.01; 12 h, $P$ < 0.01; 24 h, $P$ < 0.005), 5 μM EMP (6 h, $P$ < 0.01; 12 h, $P$ < 0.005; 24 h, $P$ < 0.005), 10 μM EMP (6 h, $P$ < 0.01; 12 h, $P$ < 0.005; 24 h, $P$ < 0.001)] (Figure 4).

### DISCUSSION

### Estramustine phosphate and malignant glioma

EMP, an antimicrotubule agent, is a steroid-alkylating agent, consisting of an oestradiol-17β-phosphate conjugated to nor-$HN_2$ through a carbamate ester linkage (Morage et al, 1992). This drug has been used against advanced prostate carcinomas as an oral agent. It was designed to allow direct delivery of the alkylating agent to the cancer cell through binding of the hormonal moiety to oestrogen receptors on cancer cells (Speicher et al, 1994). Activity against glioblastoma has been reported recently (von-Schöultz et al, 1988; 1989; 1990; 1991a,b; Piepmeier et al, 1993), although glioblastomas rarely express oestrogen-binding receptors on their cell membrane (von-Shoultz et al, 1990). EMP binds specifically to estramustine binding protein (EMBP), which is found in greater quantities in glioma tissue than in normal brain tissue (von-Shoultz et al, 1991*a*). EMBP causes a partial disassembly of microtubules (Bergenheim et al, 1994) and withdrawal of microtubules from the cell periphery towards the perinuclear area, resulting in arrest of the cell cycle in the $G_2$/M phase (Yoshida et al, 1994*b*). The distribution of EMBP may contribute to the selective cytotoxic effect of EMP on glioblastoma cells. The specific distribution of microtubule-associated proteins (MAP) in gliomas and normal brain and relationship with EMBP has not yet been clarified. The disassembling of microtubules increases the radiosensitivity of glioma cells (Ryu et al, 1994; Yoshida et al, 1994*b*) and results in a concentration-dependent alteration in cell size and shape within minutes (Piepmeier et al, 1993), as well as inhibiting proliferation and cell viability (Yoshida et al, 1996). The aim of the current study was to provide additional information about the mechanism of action of EMP. We examined how the secretion of MMPs is modulated by EMP.

© Cancer Research Campaign 1998

### Invasion of gliomas and matrix metalloproteinases

The invasiveness of glioblastomas is an essential function to overcome clinically as it is one of the key features that makes gliomas resistant to surgical resection. Matrix metalloproteinases are known to play a crucial role in the invasive nature of a number of neoplasms (Birkedal-Hansen et al, 1993), including malignant gliomas (Reict and Rucklidge, 1992). This family of zinc-dependent endopeptidases includes interstitial collagenases (MMP-1 and -8), type IV collagenases/gelatinases (MMP-2 and -9), stromelysins (MMP-3 and -10) and matrilysin (MMP-7) (Nakano et al, 1995). They are encoded by separate genes, but share some protein sequence homology and activation mechanisms (Birkedal-Hansen et al, 1993). Recent studies have disclosed that the proenzymes of MMP-2 (72 kDa) and MMP-9 (92 kDa) are activated in the presence of zinc ions to their activated forms, MMP-2 (62 kDa) and MMP-9 (83 kDa), in the extracellular space (Birkedal-Hansen et al, 1993; Nakagawa et al, 1994; Nakano et al, 1995). The molecular weight of the transparent band seen in the zymograph in this study was 62 kDa, consistent with activated MMP-2. MMP-2 and MMP-9 (type IV collagenases) are regarded as key factors in various pathological conditions, involving tissue remodelling and morphogenesis (Rao et al, 1993). Several authors have analysed the activity of MMPs in human brain tumours, compared with that in normal brain tissue (Nakagawa et al, 1994). Cell invasiveness is positively correlated with the presence of MMP-2 or MMP-9. Nakano and colleagues (1995) have quantitated the expression of MMP mRNA in human glioma cells, and have shown that U87MG cells predominantly express mRNA for MMP-2 by Northern blot analysis. This confirms that the band seen in our zymographic analysis (Figure 2) was MMP-2. The secretion of activated MMP-2 was inhibited in a concentration-dependent manner in this study. Whether secretion of MMP-9 or the other subtypes in the MMP family would be altered by the exposure with EMP could not be examined. Stearns and colleagues (1991) described that inhibition of type IV collagenase secretion by estramustine was achieved on DU 145a cells, a prostate carcinoma cell line. In the studies, he stressed that the effect was not a result of inhibition of either protein synthesis or altered rate of type IV collagenase turnover, but a result of partial disruption of the microtubule networks by binding MAP-1A as the principal target of the drug. Other authors have discussed several factors that regulate the secretion of MMPs, such as tissue inhibitors of MMPs (TIMP), urokinase-type plasminogen and neural cell adhesion molecule (NCAM) (Mohanam et al, 1993). Furthermore, one author (Nakano et al, 1995) examined the transcriptional regulation of MMP and TIMP genes by tumour promoters, growth factors and cytokines, such as TGF-β, EGF, TNF-α, (IL)-1β, and IL-6. We are not aware of any studies on the regulation of MMPs by anti-cancer agents or by the microtubules.

In the initial stage of tumour angiogenesis, capillary endothelial cells destroy the basement membrane surrounding intact capillaries and migrate through the extracellular matrix toward the source of the angiogenic stimulus. The induction of this endothelial cell migration requires the presence of MMPs (Taylor et al, 1991). Vaithilingam has reported an increase in the activity of general proteinases and type IV collagenases (MMP-2 and -9) in the serum, associated with the growth of C6 rat glioma cells in a spheroid implantation model (Vaithilingam et al, 1992).

Nakagawa and colleagues (1994) have shown that immunoreactive MMP is found in the neovascuralized areas of brain tumours. Thus, the regulation of MMPs in the extracellular matrix could be a novel method of inhibiting tumour angiogenesis. We first determined the secretion of activated MMP-2 by U87MG by a collagenolysis assay. MMP-2 secretion was suppressed by EMP in a concentration-dependent manner. Cell migration occurring in response to an immobilized substrate, such as the extracellular matrix, is called haptotaxis. Cell invasion through such a substrate is called haptoinvasion (Djakiew et al, 1993). This haptoinvasion requires initial cell motility, and secondary penetration through the substrate by the digestion and secretion of proteolytic enzymes. Thus, the U87MG cells, with reduced MMP-2 production secondary to EMP exposure, inhibited the degradative capacity of reconstituent basement membrane, Matrigel, consisting of collagen type IV (Janiak et al, 1994). Cell invasion mediated by the degradation of collagen type IV by MMP-2 represents just one of the mechanisms involved in basement membrane penetration. Another factor which may contribute to haptoinvasion is the alterations in cell shape, mediated by microtubules, which are necessary to maintain cell motility (Rutberg and Wallin, 1993; Yoshida et al, 1996). Despite the cytotoxic effect, our previous report (Yoshida et al, 1996) showed that cell population after exposure with 0 to 10 μM EMP did not change significantly when the exposure time was within 24 h. Hence, the present study was performed with the same cell densities. Therefore, production of MMP-2 for each cell must be suppressed in the series of assays, not by the cytoreductive effect of EMP.

The chemical components of Matrigel do not match those of normal brain tissue (Paulus and Tonn, 1994). Collagen type IV, however, is a major component of both normal brain structures and of blood vessels (Santell et al, 1992). Therefore, the diminished collagenolysis and the decreased invasiveness in Matrigel demonstrated in the present study may be highly implicative of clinical activity of EMP in the patients with malignant gliomas.

### CONCLUSION

In conclusion, estramustine phosphate inhibits the secretion of matrix metalloproteinases-2 and the invasiveness of human glioblastoma cells in vitro. Primarily targeting MAP-1A protein may cause partial disruption of microtubular networks that would induce reduction of MMP-2, not as a result of altered rate of MMP-2 turnover (Stearns et al, 1991). Loss of cell locomotion may account for the inhibition of invasiveness. The invasive propensity of glioblastomas may be dependent on (a) initial cell locomotion; (b) the secretion of proteinases for remodelling the basement membrane and neighbouring structures; and (c) the regrowth of the tumour cell population after penetration through the adjacent structures. Thus, the inhibition and disruption of these processes must inhibit glioma cell invasion. The importance of additional mechanisms, such as transcriptional regulation of MMP genes and the relevance of microtubule disassembly, remain to be established.

### ACKNOWLEDGEMENT

This study was supported by a Grant-in-Aid for Scientific Research from the Ministry of Education, Science and Culture in Japan (No. 07671548).

### REFERENCES

Abe T, Mori T, Kohno K, Seiki M, Hayakawa T, Welgus HG, Hori S and Kuwano M (1994) Expression of 72 kDa type IV collagenase and invasion activity of human glioma cells. *Clin Exp Metas* **12**: 296–304

© Cancer Research Campaign 1998

Albini A, Iwamoto Y, Kleinman HK, Martin GR, Aaronson SA, Kozlowski JM and McEwan RN (1987) A rapid in vitro assay for quantitating the invasive potential of tumor cells. *Cancer Res* **47**: 3239–3245

Barna BP, Estes ML and Jacobs BA (1990) Human astrocytes proliferate in response to tumor necrosis factor alpha. *J Neuroimmunol* **30**: 239–243

Bergenheim AT, Gunnarsson PO, Edman K, Schoultz EV and Hariz M (1993) Uptake and retention of estramustine and presence of estramustine binding protein in malignant brain tumours in humans. *Br J Cancer* **67**: 358–361

Bergenheim AT, Bjork P, Bergh J, von-Schoultz E, Svedberg H and Henriksson R (1994) Estramustine-binding and specific binding protein of the anti-mitotic compound estramustine in astrocytoma. *Cancer Res* **54**: 4974–4979

Bernstein JJ, Laws ER, Levine KV, Wood LB, Tadvalkar G and Golgberg WJ (1991) C6 glioma-astrocytoma cells and fetal astrocyte migration into artificial basement membrane: A permissive substrate for neural tumors but not fetal astrocytes. *Neurosurgery* **28**: 652–658

Birkedal-Hansen H, Moore WGI, Bodden MK, Windsor LJ, Birkendal-Hansen B, DeCarlo A and Engler JA (1993) Matrix metalloproteinases: A review. *Crit Rev Oral Biol Med* **4**: 197–250

Bohn W, Roser K, Hohenberg H, Mannweiler K and Traub P (1993) Cytoskeleton architecture of C6 rat glioma cell subclones differing in intermediate filament protein expression. *J Struct Biol* **111**: 48–58

Cameron RS and Rakic P (1994) Identification of membrane proteins that comprise the plasmalemmal junction between migrating neurons and radial glial cells. *J Neurosci* **14**: 3139–3155

Coomber BL (1991) Cytoskeleton in TGF-beta- and bFGF-modulated endothelial monolayer repair. *Exp Cell Res* **194**: 42–47

Dahllof B, Billstrom A, Cabral F and Hartley-Asp B (1993) Estramustine depolymerizes microtubules by binding to tubulin. *Cancer Res* **53**: 4573–4581

Djakiew D, Pflug BR, Delsite R, Onoda M, Lynch JH, Arand R and Thompson EW (1993) Chemotaxis and chemokinesis of human prostate tumor cell lines in response to human prostate stromal cell secretory proteins containing a nerve growth factor-like protein. *Cancer Res* **53**: 1416–1420

Hudes GR, Greenberg R, Krigel RL, Fox S, Scher R, Litwin S, Watts P, Speicher L, Tew K and Comis R (1992) Phase 2 study of Estramustine and Vinblastine, two microtubule inhibitors, in hormone-refractory prostate cancer. *J Clin Oncol* **10**: 1754–1761

Janiak M, Hashmi HR and Janowska-Wieczorek A (1994) Use of the matrigel-based assay to measure the invasiveness of leukemic cells. *Exp Hematol* **22**: 559–565

Lin CH and Forscher P (1993) Cytoskeleton remodeling during growth cone-target interactions. *J Cell Biol* **121**: 1369–1383

Maria BL, Cumming R and Sukhu L (1992) Crown of microfilaments in the extending cytoplasmic processes of medulloblastoma glial progenitors. *Ca J Neurol Sci* **19**: 23–33

Mohanam S, Sawaya R, McCutcheon I, Ali-Osman F, Boyd D and Rao JS (1993) Modulation in vitro of human glioblastoma cells by urokinase-type plasminogen activator receptor antibody. *Cancer Res* **53**: 4143–4147

Moraga D, Rivas-Berrios A, Farías G, Wallin M and Maccioni RB (1992) Estramustine-phosphate binds to a tubulin binding domain on micro-tubule-associated proteins MAP-2 and tau. *Biochim Biophys Acta* **1121**: 97–103

Nakagawa T, Kubota T, Kabuto M, Sato K, Kawano H, Hayakawa T and Okada Y (1994) Production of matrix metalloproteinases and tissue inhibitor of metalloproteinases-1 by human brain tumors. *J Neurosurg* **81**: 69–77

Nakano A, Tani E, Miyazaki K, Yamamoto Y and Furuyama J (1995) Matrix metalloproteinases and tissue inhibitor of metalloproteinases in human gliomas. *J Neurosurg* **83**: 298–307

Paulus W and Tonn JC (1994) Basement membrane invasion of glioma cells mediated by integrin receptors. *J Neurosurg* **80**: 515–519

Pedersen PH, Ness GO, Engebraaten O, Bjerkvig R, Lillehaug JR and Laerum OD (1994) Heterogenous response to the growth factors [EGF, PDGF (bb), TGF-alpha, bFGF, IL-2] on glioma spheroid growth, migration, and invasion. *Int J Cancer* **56**: 255–261

Piepmeier JM, Keefe DL, Weinstein MA, Yoshida D, Zielinski J, Lin TT, Chen Z and Naftolin F (1993) Estramustine and estrone analogs rapidly and reversibly inhibit deoxyribonucleic acid synthesis and alter morphology in cultured human glioblastoma cells. *Neurosurgery* **32**: 422–431

Rao J, Steck PA, Mohanan S, Stetler-Stevenson WG, Liotta LA and Sawaya R (1993) Elevated level of *Mr* 92,000 type IV collagenase in human brain tumors. *Cancer Res* **53**: 2208–2211

Reict A and Rucklidge GJ (1992) Invasion of brain tissue by primary glioma: Evidence for the involvement of urokinase-type plasminogen activator as an activator of type IV collagenase. *Biochem Biophys Res Commun* **186**: 348–354

Rutberg M and Wallin M (1993) Estramustine induces disorganization of microtubules, perinuclear retraction of vimentin and endoplasmatic reticulum, and inhibits cell migration. *Acta Histochem* **95**: 155–167

Ryu S, Gabel M, Khil MS, Lee YJ, Kim SH and Kim JH (1994) Estramustine: a novel radiation enhancer in human carcinoma cells. *Int J Radiat Oncol Biol Phys* **30**: 99–104

Santell L, Marotti K, Bartfeld NS, Baynham P and Levin EG (1992) Disruption of microtubules inhibits the stimulation of tissue plasminogen activator expression and promotes plasminogen activator inhibitor type 1 expression in human endothelial cells. *Exp Cell Res* **201**: 358–365

Speicher LA, Laing N, Barone LR, Robbins JD, Seamon KB and Tew KD (1994) Interaction of an estramustine photoaffinity analogue with cytoskeletal proteins in prostate carcinoma cells. *Mol Pharmacol* **46**: 866–872

Stearns ME, Wang M and Sousa O (1991) Evidence that estramustine binds MAP-1 A to inhibit type IV collagenase secretion. *J Cell Sci* **98**: 55–63

Taylor CM, Weiss JB and Lye RH (1991) Raised levels of latent collagenase activating angiogenesis factor (ESAF) are present in actively growing human intracranial tumours. *Br J Cancer* **64**: 164–168

Vaithilingam SI, McDonald W, Brown NK, Stroude E, Cook RA and DelMaestro RF (1992) Serum proteolytic activity during the growth of C6 astrocytoma. *J Neurosurg* **77**: 595–600

von-Schoultz E, Lundblad D, Bergh J, Grankvist K and Henriksson R (1988) Estramustine binding protein and anti-proliferative effect of estramustine in human glioma cell lines. *Br J Cancer* **58**: 326–329

von-Schoultz E, Gunnarsson P and Henriksson R (1989) Uptake, metabolism and antiproliferative effect of estramustine phosphate in human glioma cell lines. *Anticancer Res* **9**: 1713–1716

von-Shoultz E, Lundgren E and Henriksson R (1990) Effects of estramustine and its constituents on human malignant glioma cells. *Anticancer Res* **10**: 693–696

von-Shoultz E, Begenheim T, Grankvist K and Henriksson R (1991*a*) Estramustine binding protein in human brain-tumor tissue. *J Neurosurg* **74**: 962–964

von-Shoultz E, Grankvist K, Gustavson H and Henriksson R (1991*b*) Effects of estramustine on DNA and cell membrane in malignant glioma cells. *Acta Oncol* **30**: 719–723

Yoshida D, Cornell-Bell A and Piepmeier JM (1994*a*) Selective antimitotic effects of estramustine correlate with its antimicrotubule properties on glioblastoma and astrocytes. *Neurosurgery* **34**: 863–868

Yoshida D, Piepmeier JM and Weinstein M (1994*b*) Estramustine sensitizes human glioblastoma cells to irradiation. *Cancer Res* **54**: 1415–1417

Yoshida D, Piepmeier JM and Teramoto A (1996) In vitro inhibition of cell proliferation, viability, and invasiveness in U87MG human glioblastoma cells by estramustine phosphate. *Neurosurgery* **39**: 360–366

© Cancer Research Campaign 1998